# The Impact of Medical Physical Training and a Structured Personalized Exercise Training Program on Hemodynamic Parameters and Arterial Stiffness in Pregnant Women

**DOI:** 10.3390/biomedicines12050986

**Published:** 2024-04-30

**Authors:** Izabella Petre, Stela Iurciuc, Florina Buleu, Ion Petre, Radu Dumitru Moleriu, Daian Popa, Vladiana Turi, Anca Bordianu, Rabia Tasdemir, Laura Maria Craciun, Luciana Marc, Flavia Mirela Barna, Mircea Iurciuc

**Affiliations:** 1 Department XII of Obstetrics and Gynaecology, “Victor Babes” University of Medicine and Pharmacy, 300041 Timisoara, Romania; petre.izabella@umft.ro; 2 County Emergency Clinical Hospital “Pius Brinzeu”, 300732 Timisoara, Romania; florina.buleu@umft.ro (F.B.); rabia.tasdemir@gmail.com (R.T.); 3 Department of Cardiology, “Victor Babes” University of Medicine and Pharmacy, 300041 Timisoara, Romania; maria-laura.craciun@umft.ro (L.M.C.); iurciuc.mircea@umft.ro (M.I.); 4 Doctoral School, “Victor Babes” University of Medicine and Pharmacy, 300041 Timisoara, Romania; petre.ion@umft.ro (I.P.); daian-ionel.popa@umft.ro (D.P.); 5 Department of Functional Sciences, Medical Informatics and Biostatistics Discipline, “Victor Babes” University of Medicine and Pharmacy, 300041 Timisoara, Romania; radu.moleriu@umft.ro; 6 Department of Surgery, Emergency Discipline, “Victor Babes” University of Medicine and Pharmacy, 300041 Timisoara, Romania; 7 Department of Internal Medicine, “Victor Babes” University of Medicine and Pharmacy, 300041 Timisoara, Romania; turi.vladiana@umft.ro; 8 Department of Plastic Surgery and Reconstructive Microsurgery Bagdasar-Arseni, Emergency Hospital Bucharest, University of Medicine and Pharmacy “Carol Davila”, 010825 Bucharest, Romania; anca.bordianu@gmail.com; 9 Centre for Molecular Research in Nephrology and Vascular Disease, Faculty of Medicine, “Victor Babes” University of Medicine and Pharmacy, 300041 Timisoara, Romania; marc.luciana@umft.ro; 10 Department of Internal Medicine II, “Victor Babes” University of Medicine and Pharmacy, 300041 Timisoara, Romania; 11 Department of Finance, Faculty of Economics and Business Administration, West University of Timisoara, 300115 Timisoara, Romania; flavia.barna@e-uvt.ro

**Keywords:** personalized exercise training program, arterial stiffness, pulse wave velocity, augmentation index, pregnancy, cardiovascular risk

## Abstract

Introduction: In developed countries, heart disease is the primary cause of maternal mortality during pregnancy. Arterial stiffness, an independent risk factor for atherosclerosis and a predictor of cardiovascular complications, can be assessed using the augmentation index (AIx) and pulse wave velocity (PWV). In this prospective study, we aimed to evaluate diverse hemodynamic parameters and arterial stiffness in pregnant women before and after participating in a structured, personalized exercise training program. Materials and methods: Forty healthy pregnant women, non-smokers, who agreed to participate daily for 12 weeks in a physical exercise training program under the supervision of a team made up of an obstetrician, a cardiologist, and a physiotherapist were included. Anthropometric characteristics, arterial function, and physical activity data were collected from the participants at two different time points: at the beginning of the exercise training program (T0) and at the end, after 12 weeks (T1). Results: Upon conducting a statistical analysis, it was discovered that there were noteworthy disparities (*p* = 0.05) in body mass index, brachial AIx, systolic blood pressure, and pulse pressure values between the two time points. The regression analysis for the AIx brachial values and the PWVao values from Trim II (T0) and Trim III (T1) showed major differences between these two time points; the association between the AIx brachial values in the second and third trimesters of pregnancy revealed a strong direct significant correlation (*p* < 0.001), and the correlation between the PWVao values in the second (T0) and third trimester (T1) of pregnancy was weak and insignificant (*p* = 0.12). Conclusions: The findings of our study indicate that a personalized exercise training program positively impacts the physical and psychological well-being of pregnant women, leading to a reduction in PWV.

## 1. Introduction 

In addition to traditional risk factors for cardiovascular disease, women face an extra burden of gender-specific risk factors. Milestones in a woman’s reproductive life history can influence or reveal short- and long-term cardiometabolic and cardiovascular pathways [1]. 

During pregnancy, the maternal cardiovascular system undergoes necessary adjustments to accommodate physiological changes. Normal pregnancy is characterized by significant increases in plasma volume and cardiac output, primarily due to arterial vasodilation. These adaptations play a crucial role in ensuring a successful pregnancy. The expansion of the aorta is linked to the arterial vasodilation that occurs during pregnancy [2]. It was also demonstrated that the physiological increase in cardiac output and circulating blood volume could secondarily increase the value of measures of arterial stiffness [3]. Research has shown that elevated arterial stiffness during pregnancy contributes to the development of conditions like pregnancy-induced hypertension and fetal growth restriction [4]. But, also in a study where 541 healthy normotensive pregnant women were analyzed, no significant changes in arterial stiffness were observed as pregnancy progressed [5]. 

Throughout normal pregnancy, the body experiences a marked increase in vascular volume and cardiac output to meet its metabolic demands. However, in this situation, low aortic stiffness prevents substantial increases in pulse pressure (PP) and systolic blood pressure (SBP). In individuals who are not pregnant, arterial stiffness typically intensifies as one moves from the aorta to the peripheral arteries, which is essential for minimizing the energy carried by the forward wave to the microcirculation [6]. Over the years, many studies have associated the severity and occurrence of arterial stiffness with various pathologies with cardiovascular impact [7] or even with health outcomes on longer-term follow-up [8]. Studies have shown that aortic stiffness is consistently elevated in pregnancy-associated hypertension compared to healthy pregnant women [9].

There is growing evidence that exercise improves systemic endothelial function and arterial stiffness in a variety of patients, from children to older adults, and in a variety of diseases or as a preventive therapy for gestational hypertensive disorders [10,11]. Surprisingly, the effects of exercise on the vasculature of healthy pregnant women have been poorly studied, and there are almost no data on individualized exercise programs [12]. To our knowledge, only one study has examined the effects of exercise training on endothelial function during healthy pregnancy more than a decade ago. The study used a different method of measuring arterial stiffness (flow-mediated dilation (FMD)) not the gold standard carotid–femoral pulse wave velocity (PWV), which was improved by 30% when moderate-intensity training was initiated between weeks 16 and 20 of pregnancy [13]. Regarding arterial stiffness, significant improvements in PWV were observed with prenatal exercise early after birth, but this has not been studied during pregnancy [14].

However, the impact of physical activity on arterial stiffness in healthy pregnant women, which could potentially decrease distal pulsatility and protect the microcirculatory system, remains uncertain. 

Despite the European guidelines on the management of cardiovascular disease in pregnancy [15], the implementation of strategies to improve adherence to methods to decrease pregnancy-related cardiovascular disease mortality remains sub-optimal, especially in developing countries [16]. 

Moreover, there is no global consensus on the reduction in cardiovascular risk via physical activity in pregnant women [17], or what kind of exercise can be performed by pregnant women, as there was evaluated in different cardiovascular pathologies or even in healthy subjects [18,19,20,21,22,23,24,25], especially analyzing the impact on endothelial dysfunction. 

So, this study aimed to assess the impact of a personalized exercise training program on hemodynamic parameters and arterial stiffness in pregnant women and to determine if this training regimen improves hemodynamic parameters.

## 2. Material and Methods 

### 2.1. Study Design and the Selection of Participants

This research was conducted at the Obstetrics and Gynaecology Departments of the Emergency Clinical Hospital “Pius Brînzeu” in Timisoara, involving 40 pregnant women who met the inclusion criteria for the study. These women had healthy pregnancies with only one fetus each. Before participating, all patients gave informed consent, adhering to the principles outlined in the Declaration of Helsinki [26]. This study was approved by the Ethics Committee of the Emergency Clinical Hospital “Pius Brînzeu” Timișoara (decision no. 54/20 April 2017).

Only pregnant women aged ≥18 years and ≤45 years, with gestational age in the second trimester of pregnancy, in their first pregnancy or second pregnancy, in stable clinical and hemodynamic conditions, and who expressed a desire to perform physiotherapy exercises and non-smoker were included. Pregnant women with a family history of cardiovascular disease or diagnosed before pregnancy with cardiovascular disease, aged below 18 years and more than 45 years, multiparous (more than 3 pregnancies), with hypertension or other pregnancy-induced diseases, gestational diabetes or pregnant women classified as at risk of pregnancy or who developed other pregnancy complications were excluded. 

### 2.2. Clinical and Biochemical Evaluation

Demographic information, anthropometric parameters (including age, height, weight, and body mass index (BMI)), personal and family medical history, cardiovascular risk factors, medications, and laboratory values (total cholesterol, triglycerides, complete blood count, blood glucose) were gathered at inclusion in the exercise training program by using a photometric method (Siemens Dimension RXL-MAX, Dade Behring, Erlangen, Germany). Additionally, arterial function evaluations were conducted at the beginning and end of the 12-week program. 

Blood pressure (BP) and heart rate (HR) were determined according to the European Guidelines on Cardiovascular Disease Prevention in clinical practice [27] using a manual sphygmomanometer (Riester, Jungingen, Germany), and the values obtained were compared with the values measured automatically by the Medexpert Arteriograph. 

### 2.3. Measurement of Arterial Stiffness

Using the Medexpert Arteriograph device version 3.0.0.3, created by TensioMed Kft. in Budapest, Hungary, we could non-invasively measure the pulse wave velocity (PWV). This accredited medical device provided precise readings of the central pressure pulse (PP), augmentation index (AIx), and pulse wave propagation velocity in the aorta (PWVao). To ensure the greatest accuracy, pregnant women were instructed to remain in a supine position for 10 min in a quiet room before measurements. 

The same examiner followed the determination method outlined in the expert consensus document on arterial stiffness, conducting repeated measurements. To maintain consistency, participants were required to abstain from smoking, caffeine-containing foods, or drinks for at least 3 h before the measurements. Additionally, alcohol consumption was halted at least 10 h before the investigation. As arterial diameter and stiffness exhibit circadian variation, which increases during sleep, participants were instructed not to fall asleep during the assessment. Throughout the measurements, participants refrained from speaking. 

Based on information from various sources, the standard measurement of pulse wave velocity in adult women is 7.4 m/s. However, it is essential to note that baseline values tend to be lower in young, fit women, with an average of 6.1 (ranging from 4.6 to 7.5) m/s. As women age, these values gradually increase either due to physiological changes in the vascular endothelium, increased blood pressure, or other associated pathologies, or even during pregnancy [28]. 

### 2.4. Personalized Exercise Training Program

The exercise training program was developed by a team that included an obstetrician in collaboration with a cardiologist and a physiotherapist. The included pregnant women participated daily for 12 weeks in this individualized physical training program under the supervision of a physiotherapist. 

Exercise training program exercises performed by the pregnant women created and used in this study are described in Table 1. The existence of an average age of 25 years and, consequently, an adequate physical condition allowed the use of medium-intensity exercise sets on the Borg scale in this study [29].

### 2.5. Statistical Analysis

The SPSS software (SPSS 22.0 Inc., Chicago, IL, USA) and JASP v18.3 were used for statistical analysis. Results for variables were presented as means ± standard deviation or percentages. Group comparisons were conducted using the two-tailed Student *t*-test, respectively, the Mann–Whitney test, or the ANOVA test/Kruskal–Wallis test, depending on the situation. A *p*-value of less than 0.05 was considered statistically significant. At the end of our analysis, a regression model was included to test the possible association between the studied variables; and the results were plotted using a scatter representation. The database has only numerical variables, so, we can calculate the central tendency and dispersion parameters for the entire database. For representing the data in the descriptive analysis, a boxplot or histogram chart was used. The data distribution was tested using the Shapiro–Wilk test (for a *p* value below 0.05, non-parametrical tests were applied). 

## 3. Results

### 3.1. Description of the Patient’s Lot

Forty pregnant patients in the second trimester of pregnancy were included in this study, whose mean values at inclusion in the exercise program for the variables age, height, weight, and BMI are represented in Table 2. We can say that our sample comprised young subjects, with a mean value for the age of 25 years, and women who practiced sports regularly (Table 2). 

### 3.2. Analysis of Arterial Function Parameters before and after Performing the Exercise Training Program

The analyzed pregnant women (*n* = 40) had an active life and practiced sports regularly. We gathered information about the subject’s age, height, weight, body mass index (BMI), AIx brachial values, PWVao values, systolic (Sys) and diastolic (Dia) blood pressure, and heartbeats minute in the second (at baseline, T0) and third trimester of pregnancy (after finished exercise training program, T1) (Trim II and Trim III). 

All the characteristics of arterial function variables measured with arteriography at inclusion and after performing the exercise training program are presented in the next table (Table 3).

All the characteristics of arterial function variables measured with arteriography at inclusion and after performing the exercise training program, as well as the significance obtained after applying the t-paired/Wilcoxon Signed-Rank test, are presented in Table 3. A significant improvement (*p* < 0.05) was obtained in four out of five parameters. 

**Figure 1:**
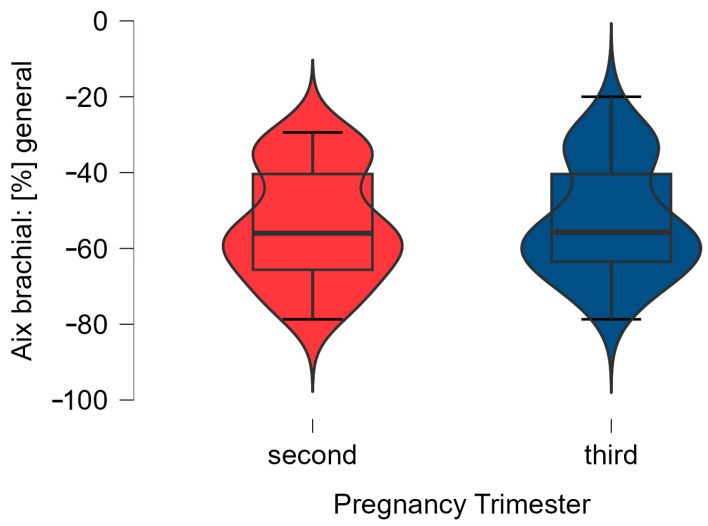
The data distribution of AIx brachial values in the second (T0) and third trimester of pregnancy (T1) (*n* = 40), using a boxplot representation with violin effect.

**Figure 2:**
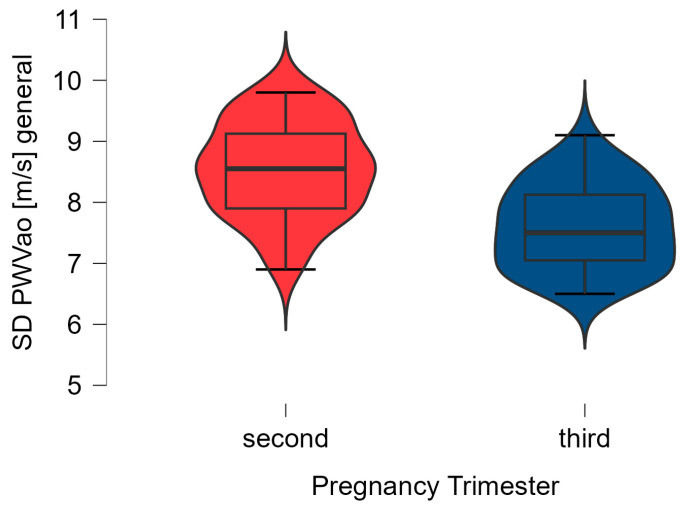
The data distribution of the PWVao values in the second (T0) and third trimester of pregnancy (T1) (*n* = 40), using a boxplot representation with violin effect.

Regarding the evolution of the AIx brachial and PWVao values, we observed a decreased in these arterial stiffness parameters in all healthy pregnant women (*n* = 40), as can be seen in Figure 1 and Figure 2. 

The regression analysis for the AIx brachial values and the PWVao values from Trim II (T0) and Trim III (T1) showed major differences between these two time points, but we also wanted to test if these variables have a relation between them. For this purpose, we have run a regression analysis in which we considered the values from Trim II as predictors and the values from Trim III as responses. The association of the sample was calculated with the Pearson coefficient. The results obtained are the following: pregnancy revealed a strong direct significant correlation between the AIx brachial values in the second and third trimesters of pregnancy $\left(r=0.97,\;\;R^{2}=0.95,\;\;p<0.001\right)$ and that the correlation between the PWVao values in the second and third trimester of pregnancy is weak and insignificant $\left(r=-0.25,\;R^{2}=0.06,\;p=0.12>0.05\right)$ . 

**Figure 3:**
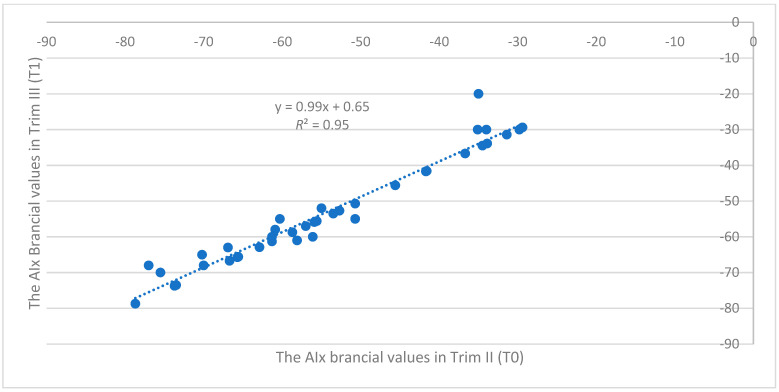
The association between the AIx brachial values in Trim II (T0) and Trim III (T1) (*n* = 40).

**Figure 4:**
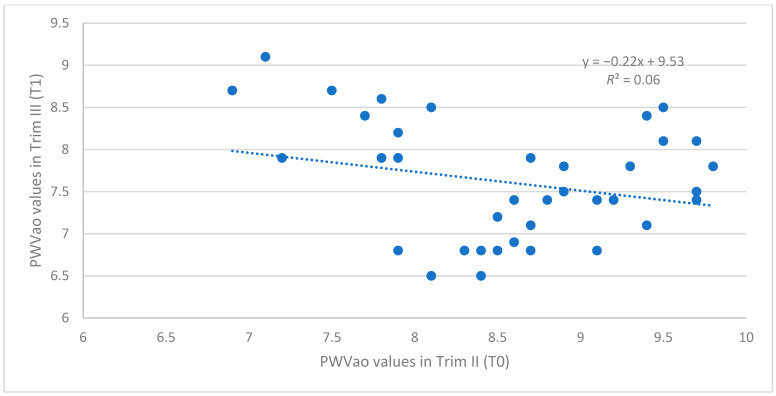
The association between the PWVao values in Trim II (T0) and Trim III (T1) (*n* = 40).

In conclusion, the AIx brachial values are constantly decreasing for the whole sample, while for PWVao values, the decreasing process does not have the same dynamic in the whole sample. This relation between the two variables is presented in Figure 3 and Figure 4.

## 4. Discussion

The present study concerns a special group of subjects, pregnant women, who, according to the guideline for the management of cardiovascular disease during pregnancy from the European Society of Cardiology, are at very high cardiovascular risk [15]. Additionally, it is essential to note that pregnancy alone is linked to a significant three to four-fold increase in the occurrence of acute myocardial infarction compared to women of the same age who are not pregnant [30,31,32]. 

The etiology of cardiovascular disease in pregnancy differs from that of the general population; most pregnant women develop coronary artery disease that has non-atherosclerotic mechanisms, followed closely by pregnancy-related spontaneous coronary artery dissection (43% pregnancy-related cardiovascular disease), and coronary arteries in pregnant women are angiographically normal [33,34]. The mechanisms of acute myocardial infarction in pregnant women with angiographically normal coronary arteries remain unclear and include transient coronary spasm [35] due to either increased vascular reactivity and/or arterial stiffness that occurs physiologically in pregnancy [28,36,37]. 

So, in this study, we examined endothelial dysfunction as a marker of subclinical arterial stiffness among healthy pregnant women and the impact of a personalized exercise training program on it. This study has three major findings. Our main finding was the reduction in PWV values with significant differences between the two times studied (T0 at inclusion in the exercise training program and T1 at the finished of the exercise training program). Secondly, the benefit of this program is reducing and normalizing cardiovascular risk factors (blood pressure, women’s weight, pulse pressure, etc.). Thirdly, the performance in the diagnosis and monitoring of endothelial dysfunction with the help of PWV measurement in pregnant women. dysfunction. Another added value of the current study is that this program was developed, initiated, and continued in a local hospital and led by a team that included an obstetrician, a cardiologist, and a physiotherapist. 

Specialty studies show how physical activity has been a subject of considerable research and attention during pregnancy and they highlight the importance of physical activity in order to promote a healthy status. An appropriate level of physical activity or an exercise program during pregnancy is very useful. Choosing to continue and maintain this health status is a recent research area in maternal and child health, and pregnant women can choose different levels of physical activity [38]. Clapp et al. found that women who continued to exercise during pregnancy maintained their training regimens and their fitness levels well above the cardiovascular risk index, and their level of risk is well below those present in both the general population and the number of women who temporarily interrupted their exercise during pregnancy. Further, they also observed that pregnant women who engage in weight-bearing exercise throughout their pregnancy preserve their overall fitness and maintain a low cardiovascular risk profile during the perimenopausal phase [39]. 

The American College of Obstetricians and Gynecologists (ACOG) released its initial findings and suggestions regarding physical activity during pregnancy in 2002. According to the ACOG, pregnant women without any medical or obstetric complications should engage in at least 30 min of moderate-intensity exercise. Conducting a thorough clinical examination before providing exercise recommendations to pregnant women is essential. Unless there are any reasons to avoid exercise, it is recommended to engage in moderate-intensity exercises regularly. Additionally, pregnant women should be educated on the signs indicating the need to stop physical training [40]. When developing a physical exercise program, it is crucial to consider the type and intensity of the exercise [41]. 

The American College of Obstetricians and Gynecologists [42], the Society of Obstetricians and Gynecologists of Canada (SOGC) [43], and the Royal College of Obstetricians and Gynecologists of Australia and New Zealand (RANZCOG) [44] guidelines were analyzed in a review [17] regarding recommending exercise during pregnancy. All these guidelines universally agreed that women who do not have any contraindications should be encouraged to engage in regular aerobic and strength-conditioning exercises during pregnancy. The ACOG recommends that pregnant women aim for 150 min of exercise per week, which can be broken down into 20–30 min per day. The RAZCOG suggests that pregnant women can aim for 150–300 or 70–150 min of vigorous exercise per week (with each session not exceeding 60 min). The SOGC does not officially recommend exercise duration during pregnancy. It is important to note that exercising during pregnancy can positively affect both the mother’s and the fetus’s health. 

At the same time, physical inactivity is considered a significant risk factor for maternal mortality. Furthermore, regular exercise during pregnancy has not been linked to adverse perinatal outcomes [45,46]. Pregnant women who adhered to the recommended physical activity guidelines of engaging in 150 min of moderate-to-vigorous physical activity per week experienced lower central and peripheral PWV levels [47].

Our research yielded a significant and noteworthy distinction in the values of systolic blood pressure, diastolic blood pressure, and heart rate when comparing two distinct periods: before and after a 12-week exercise program during pregnancy. A similar study, which involved 56 women with healthy, singleton, low-risk pregnancies (30 in the control group and 26 in the exercise group), utilized magnetocardiogram recordings to demonstrate that exercising during pregnancy can enhance cardiac autonomic control [48]. A recent systematic review and meta-analysis that investigated the effects of aerobic and resistance exercise on blood pressure during pregnancy while experiencing clinical conditions such as gestational diabetes mellitus and obesity during pregnancy observed that exercise has the potential to decrease the typical rise in blood pressure associated with these conditions. These discoveries carry significant importance for pregnant women who are at risk of developing gestational hypertension and pre-eclampsia [49]. 

The findings from our study, which demonstrate a decrease in arterial stiffness and improvements in hemodynamic parameters, align with the existing data reported in the literature. At the onset of the second trimester of pregnancy, the exercise and control groups exhibited no significant disparity in PWV values when assessing arterial stiffness. However, upon analyzing the same group of pregnant women one month after delivery, those who engaged in regular exercise throughout their pregnancy displayed a significantly lower PWV compared to their second-trimester measurements (1145.9 ± 88.1 cm/s vs. 1122.7 ± 100.2 cm/s, respectively; insignificant). Conversely, the control group, which did not partake in exercise, maintained a PWV of 1116.7 ± 87.9 cm/s, indicating an increase in arterial stiffness [14]. In a separate study, 191 women with high-risk singleton pregnancies were examined to assess the predictive capabilities of arterial stiffness parameters in early pre-eclampsia compared to peripheral blood pressure, angiogenic biomarkers, and uterine artery Doppler. Within this group of expectant mothers, a total of 7.3% experienced the development of pre-eclampsia. The likelihood of pre-eclampsia increased by 64% (*p*  <  0.05) for every one m/s rise in first trimester PWV, while a 1-millisecond increase in time to wave reflection resulted in an 11% decrease in the odds of pre-eclampsia (*p*  <  0.01). The researchers assert that arterial stiffness is a superior predictor of pre-eclampsia compared to other analyzed methods, demonstrating its early detection and heightened efficacy [50]. 

The regression analysis for the AIx brachial values and the PWVao values from Trim II (T0) and Trim III (T1) showed major differences between these two time points as follows: pregnancy revealed a strong direct significant correlation between the AIx brachial values in the second and third trimester of pregnancy $\left(r=0.97,\;R^{2}=0.95,\;p<0.001\right)$ and that the correlation between the PWVao values in the second and third trimester of pregnancy is weak and insignificant $\left(r=-0.25,\;R^{2}=0.06,\;p=0.12\right)$ . 

While our study highlights the advantages of personalized exercise training programs for healthy pregnant women in their second trimester, there is still room for improvement. It is crucial to address the identified gaps and take action to enhance general exercise programs for pregnant women, regardless of their cardiovascular risk factors. Various barriers hinder the implementation of these exercise programs, and we will focus on a few encountered in our study. These include patients’ willingness to participate, inequalities in access and availability of exercise facilities, and the need for more collaboration between obstetricians, cardiologists, and physiotherapists, possibly due to limited experience [51,52]. 

Initiating prevention measures for cardiovascular disease is crucial at the earliest stages for individuals of all genders. We believe that women in particular should take advantage of cardiac consultations during pregnancy, perimenopause, and menopause to evaluate their cardiovascular risk. It is widely known that early onset of menopause is associated with an increased likelihood of developing cardiovascular disease, making it a significant red flag during medical check-ups [1]. One method to identify potential major adverse cardiac events (MACEs) is via screening mammography, which can assess breast density in overweight women and serve as a predictive tool [53]. Indeed, tissue over-expressed inflammatory cytokines and oxidative stress molecules leading to cardiac dysfunction and worse prognosis were observed [54]. Overweight and metabolic distress could cause MACEs in women via over-inflammation [55]. 

### Limitations of the Study

One limitation of this study is its reliance on data collected from a single regional hospital in Romania, which restricts the generalizability of the findings to other contexts due to variations in healthcare policies [51]. However, it is important to note that the study’s focus on healthy pregnant women, who are the majority of the pregnant population, ensures the relevance of the findings to a significant portion of the healthcare community. Another constraint is the lack of homogeneity in the age distribution of the analyzed group, as only pregnant women under the age of 40 were included, with a significantly higher proportion being under the age of 30 also the sample size, which is quite small, underlining the need for future studies with a larger sample to support the results of this research.

By excluding pregnant women who possess any of the numerous high-risk factors that have the potential to impact arterial stiffness, selection bias is effectively minimized. Consequently, the chances of encountering selection bias are meager, enabling the normal ranges we have delineated to apply to low-risk women across different populations. 

The exclusion of pregnant women with any of a long list of high-risk factors (which could affect arterial stiffness) should effectively reduce selection bias. As a result, the likelihood of encountering selection bias is minimal, allowing the normal ranges we outline to be relevant for low-risk women in various populations. It is also recognized that the accuracy of all self-reported information could not be fully verified in terms of perceived correct exercise intensity (intensity perception may differ from one pregnant woman to another), but we believe that we have eliminated these reasons by objectively tracking the warning signs for discontinuation of physical activity (see Table 1). 

## 5. Conclusions

Our study results showed that physical training, coordinated by a medical team that consisted of an obstetrician, a physiotherapist, and a cardiologist, improves the hemodynamic parameters (SBP, PP, and HR) of the healthy pregnant woman during pregnancy, and that the PWV is a reliable method to measured arterial stiffness in this type of patients. Strictly supervised physical training may reduce pregnancy-induced hypertension. 

Additionally, regular exercise in healthy pregnant women decreased arterial stiffness, suggesting that regular exercise may help prevent hypertensive disorders during pregnancy and that our personalized exercise training program may be used to reduce blood pressure and prevent the development of arterial rigidity during pregnancy. We believe that this personalized exercise training program and, moreover, assessment by a multidisciplinary medical team represents an opportunity for the early detection of risk factors, refinement of CVD risk assessment, and implementation of primary prevention in women.

However, further studies are required to determine whether this adaptation to exercise can effectively reduce the risk of adverse outcomes associated with gestational conditions characterized by impaired autonomic control, such as diabetes, hypertension, pre-eclampsia, and excessive weight gain.

## Figures and Tables

**Table 1:** Description of the physical exercises performed during the intervention program.

Position	Exercise	Sets	Repetitions
Standing up	Circular movements of the head to the left and to the right	1	10
Standing up	Hands on the shoulders, raise arms and inhale, exhale on bringing hands back to the shoulders	1	10
Standing up	Legs pulled apart, with a straight back, both hands on hips, turn the trunk to the left, and then turn the trunk to the right and exhale	1	10
Standing up	Back straight, arms extended forward, heels on the floor, bend knees, extend knees, and exhale.	2	10 (1 min break between the series)
Standing up	With 2-kg weights in hands, do alternative back and forth lunges.	2	10 (1 min break between the series)
Standing up	With 2-kg weights in hands, bring the arm up to 90 degrees and exhale slowly when bringing the arm back down.	2	10 (1 min break between the series)
Standing up	With 2-kg weights in hands, flex forearms on the arms; let the air out slowly as you bring forearm back down.	2	10 (1 min break between the series)
Standing up	With arms raised at 90 degrees and elbows bent, bring arms together in front of you, and then take arms back and exhale	1	10
Standing up	Extend arms forward as you lift up each leg out laterally and exhale	1	10
Sitting on a fitness ball	With 2-kg weights attached to the ankles, lift each leg and inhale, bring leg back down slowly, and exhale.	1	10 for each leg
Sitting on a fitness ball	Using a strip, bring leg to the back, eyes following the arm, bring leg in the initial position, and exhale	1	10 for each leg
Sitting on a fitness ball	With both hands-on hips, rotate the trunk laterally	1	10 for each leg
Warning signs to stop physical activity during pregnancy	Vaginal bleeding, shortness of breath, dizziness, headache, precordial chest pain, easy fatigability, target heart rate > 145 bpm, decreased fetal movement, uterine contractions, pain in the lower back, abdomen, or pelvic area (potentially indicating pre-term labor)		

**Table 2:** Descriptive data on pregnant women at baseline (T0, *n* = 40).

UPN	AGE (Years)	Pregnancy	Cardiovascular Risk Factors	Medical History	At Baseline (T0)			
					Gestational Week	Height (cm)	Weight (cm)	BMI (kg/m^2^)
#1	18	G1P0	No	No	15 weeks 5 days	155	40	16.6
#2	19	G1P0	No	No	23 weeks 4 days	169	67	23.5
#3	19	G1P0	No	No	16 weeks 5 days	169	75	26.3
#4	19	G1P0	No	No	19 weeks 4 days	170	59	20.4
#5	19	G1P0	No	No	20 weeks 2 days	164	58	21.6
#6	20	G1P0	Obesity	No	15 weeks 3 days	160	88	34.4
#7	20	G1P0	No	No	16 weeks 6 days	175	65	21.2
#8	20	G1P0	No	No	21 weeks 5 days	167	65	23.3
#9	20	G1P0	No	No	18 weeks 5 days	152	54	23.4
#10	20	G2P1	No	No	16 weeks 5 days	155	69	28.7
#11	21	G1P0	No	No	23 weeks 3 days	169	76	26.6
#12	21	G1P0	No	No	22 weeks 6 days	157	73	29.6
#13	21	G1P0	No	No	18 weeks 6 days	161	75	28.9
#14	21	G1P1	No	No	16 weeks 5 days	170	81	28
#15	21	G1P0	No	No	19 weeks 4 days	168	75	26.6
#16	21	G1P0	No	No	21 weeks 1 days	164	69	25.7
#17	21	G1P0	No	No	20 weeks 4 days	162	98	37.3
#18	21	G1P0	No	No	16 weeks 2 days	167	52	18.6
#19	21	G1P0	No	No	14 weeks 6 days	168	68	24.1
#20	22	G1P0	No	No	20 weeks 7 days	185	87.5	25.5
#21	22	G1P0	Obesity	No	22 weeks 6 days	178	101	31.9
#22	22	G1P0	No	No	23 weeks 5 days	168	81	28.7
#23	22	G1P0	No	No	17 weeks 5 days	160	69	27
#24	23	G1P0	Obesity	No	20 weeks 4 days	165	96	35.3
#25	24	G1P0	No	No	16 weeks 3 days	168	78	27.6
#26	24	G1P0	No	No	16 weeks 5 days	164	68	25.3
#27	24	G1P0	Obesity	No	20 weeks 3 days	160	98	38.3
#28	25	G1P0	No	No	19 weeks 6 days	168	78	27.6
#29	26	G1P0	Obesity	Cholecystectomy	17 weeks 4 days	168	97	34.4
#30	27	G1P0	No	No	22 weeks 1 day	164	68	25.3
#31	27	G1P0	No	No	16 weeks 6 days	167	73	26.2
#32	28	G1P0	Obesity	No	20 weeks 4 days	151	74	32.5
#33	28	G1P0	No	No	18 weeks 3 days	158	51	20.4
#34	28	G1P0	No	No	17 weeks 5 days	172	65	22
#35	30	G1P0	No	Appendectomy	21 weeks 2 days	165	61	22.4
#36	35	G1P0	No	No	19 weeks 5 days	168	68	24.1
#37	35	G1P0	No	No	16 weeks 5 days	185	87.4	25.5
#38	43	G1P0	No	Chronic Gastritis	17 weeks 6 days	164	61	22.7
#39	44	G2P1	No	No	18 weeks 5 days	152	55	23.5
#40	45	G1P0	No	No	20 weeks 2 days	169	64	22.2

UPN, unic patient number.

**Table 3:** Arterial function parameters measure with arteriograph at T0 and T1 (*n* = 40); in the case where the data are normally distributed, a parametrical test was applied—the *t*-paired test (*), respectively, where the data are not normally distributed, a non-parametrical test was applied—the Wilcoxon Signed-Rank test (**). The level of significance is α=0.05 . The significant results are highlighted in grey.

Statistics	AIx Brachial [%]		PWVao [m/s]		SBP [mmHg]		DBP [mmHg]		HR [bpm]	
	T0	T1	T0	T1	T0	T1	T0	T1	T0	T1
*p* Value	*p* = 0.02 (**)		*p* < 0.001 (*)		*p* = 0.001 (**)		*p* = 0.006 (**)		*p* = 0.095 (**)	
Mean	−53.94	−52.66	8.54	7.62	120.55	117.80	72.18	70.33	72.63	71.55
Standard Error	2.33	2.37	0.12	0.11	2.16	1.79	1.66	1.61	1.42	1.27
Median	−56	−55.75	8.55	7.5	123	120	69.5	68.5	72.5	71
Mode	−61.3	−30	7.9	6.8	124	120	66	60	75	62
Standard Deviation	14.77	14.98	0.77	0.69	13.65	11.30	10.52	10.20	9.01	8.03
Sample Variance	218.01	224.48	0.59	0.48	186.41	127.75	110.76	104.02	81.11	64.41
Range	49.3	58.7	2.9	2.6	52	45	45	35	36	33
Minimum	−78.7	−78.7	6.9	6.5	96	100	53	55	57	57
Maximum	−29.4	−20	9.8	9.1	148	145	98	90	93	90
Count	40	40	40	40	40	40	40	40	40	40

## Data Availability

The data and materials can be provided upon reasonable request to the corresponding author.
